# A Case Series Demonstrating the Feasibility of Microdosing as a Bridging Method to Enable a Comfortable and Effective Transition From Prescribed Methadone to Buprenorphine ORT (Opiate Replacement Therapy), Without Initial Methadone Dose Reduction or Cessation, in UK Community Addiction Settings

**DOI:** 10.1192/bjo.2025.10772

**Published:** 2025-06-20

**Authors:** Zelda Summers, Jennie Rankine, Stuart Fisher

**Affiliations:** Cardiff and Vale University Health Board, Cardiff, United Kingdom

## Abstract

**Aims:** For patients prescribed methadone wishing to access buprenorphine ORT, the current practice is to reduce the methadone dose to 30/40 ml and cessation of methadone for 48 hrs prior to the switch. This exposes these patients to risk of psychological, physical distress or relapse into non-prescribed opiate use.

Using guidance published by the Canadian Journal of Addiction we adapted this to a local UK setting to evaluate its implementation into regular service provision.

We aim to demonstrate effective transition, in a series of patients prescribed methadone onto sl (sublingual) buprenorphine, without adverse effects/precipitated withdrawal or the requirement of methadone dose reduction nor 48 hour abstinence from methadone, in community-based settings.

**Methods:** We collected outcomes on 12 patients (dose range 26 ml – 90 ml of methadone daily), 12 of whom successfully transitioned from methadone to buprenorphine ORT via buprenorphine Microdosing Bridging Method.

We enquired and assessed for precipitated opiate withdrawal symptoms using a daily modified SOWS/COWS and psychological response throughout the transition period.

Patients were initiated onto buprenorphine initially using transdermal 20 mcg/24 hr buprenorphine patches over the first 48 hrs of treatment, after which urine was tested. Once the urine screen showed buprenorphine, oral initiation of sublingual buprenorphine began on Day 1 at 400 mcg daily and increased according to the following schedule:

Day 2: Usual dose methadone daily, buprenorphine 0.8 mg.

Day 3: Usual dose methadone daily, buprenorphine 1.2 mg.

Day 4: Usual dose methadone daily, buprenorphine 1.6 mg.

Day 5: Usual dose methadone daily, buprenorphine 2.0 mg.

Day 6: Usual dose methadone daily, buprenorphine 4 mg daily.

Day 7: Usual dose methadone daily, buprenorphine 6 mg daily.

Throughout this period, the patients’ methadone dose remained at their pre-transition dose. The last dose of methadone was administered when the patients were administered 6 mg sl buprenorphine daily on Day 6. The following day, patients were administered 8 mg sl buprenorphine with subsequent dose titration to achieve optimal daily dose.

**Results:** 12 patients achieved the transition from methadone to buprenorphine without experience of precipitated opiate withdrawal or adverse psychological effect.

**Conclusion:** Transition from methadone to sl buprenorphine without requiring prior dose cessation or reduction of methadone up to 90 ml daily is safe, effective and practical in a community-based and acute care setting. This provides methadone ORT patients the option of accessing buprenorphine ORT over a short period of time (10–14 days) without the experience of precipitated opiate withdrawal, and can be undertaken in a variety of community settings.

